# Improvement of laryngoscopic view by hand-assisted elevation and caudad traction of the shoulder during tracheal intubation in pediatric patients

**DOI:** 10.1038/s41598-018-37770-6

**Published:** 2019-02-04

**Authors:** Jin Hee Ahn, Doyeon Kim, Nam-su Gil, Yong Hun Son, Bong Gyu Seong, Ji Seon Jeong

**Affiliations:** Department of Anesthesiology and Pain Medicine, Samsung Medical Center, Sungkyunkwan University School of Medicine, Seoul, Korea

## Abstract

Pediatric patients have large heads and relatively small bodies, making it difficult to perform intubation even in the sniffing position. Therefore, this study was planned on the assumption that hand-assisted elevation and caudad traction of the shoulder (HA-ECTS) would compensate for the laryngoscopic view. In this observational study, 45 pediatric patients aged 0–36 months with an ASA physical status of I-III and scheduled for elective surgery under general anesthesia were enrolled. HA-ECTS was defined as hand-assisted personalized traction in the upper and caudad directions with both hands under the lower cervical area. The POGO (percentage of glottis opening) score, MO (mouth opening), and LHS (laryngoscopic handling score) were compared before and after HA-ECTS. The median [range] POGO score was 30[10–50]% and 60[15–80]% before and after HA-ECTS, respectively (median difference, 20; 95% confidence interval [CI] 10 to 25%; P = 0.002). MO was 1.0[0.8–1.9] cm and 1.8[1.3–2.0] cm before and after HA-ECTS, respectively (median difference, 0.45 cm; 95% CI 0.25 to 0.60; P < 0.001). The ease of laryngoscopic handling was improved after HA-ECTS(P < 0.001). The application of HA-ECTS to pediatric patients younger than 3 years improved POGO score, MO, and LHS and could prove to be an assistive technique for tracheal intubation.

## Introduction

In pediatric patients in the supine position, reduction of airway tone during general anesthesia leads to retraction of the tongue to the posterior pharyngeal wall, resulting in collapse of the upper airway and limitation in advancement of the endotracheal tube and laryngoscopic view^1,2^. If proper positioning and adequate laryngoscopic view for tracheal intubation are not obtained, intubation becomes difficult, and intubation time is prolonged. Improvement of the laryngoscopic view and position is important when performing tracheal intubation in pediatric patients.

The sniffing position (SP) is recommended during tracheal intubation in pediatric patients as the standard position of glottis exposure with alignment of the external auditory meatus and sternal notch (AES)^3–5^. Younger pediatric patients do not require head elevation to obtain the SP because their large heads and small chests allow for optimal visualization in a flat position with mild extension and no additional head elevation^6–8^. Generally, optimal intubation positioning involves the SP in pediatric patients above 2 years of age. For pediatric patients under 2 years of age, head extension without elevation and with or without shoulder elevation results in proper intubating conditions^6–9^. However, the direct laryngoscopic view is not improved in every pediatric patient in the SP, and it is necessary to improve actual visualization by considering the relationship with oropharyngeal structures as well as ideal anatomic axes. Moreover, Lee *et al*. reported that the laryngeal axes and line of vision improved by moving the laryngeal structure in the caudal direction through gravity in the back-up position^10^. Therefore, we performed hand-assisted elevation and caudad traction of the shoulder (HA-ECTS) so that laryngeal exposure would be improved due to increase of space in oropharyngeal cavity and improvement in laryngeal axes and line of vision in tracheal intubation in pediatric patients. We hypothesized that HA-ECTS improves the laryngoscopic view and handling in pediatric patients. The aim of our study was to confirm the improvement degree of the laryngoscopic view after HA-ECTS in pediatric patients under 36 months of age.

## Methods

### Ethics

The study protocol was approved by the Institutional Review Board (Samsung Medical Center, South Korea, IRB No. 2018–03–132, May 11, 2018) and the study design was registered in the Clinical Trial Registry of Korea (KCT 0003020, July 24, 2018). Methods of the study were carried out in accordance with the relevant guidelines and regulations. Written informed consent was obtained from the guardians of the pediatric patients.

### Study design and subjects

This prospective and observational study was including Pediatric patients aged less than 36 months with an ASA physical status of I-III and scheduled for elective surgery under general anesthesia were enrolled in this study from May 2018 to August 2018. Patients with head and neck malformation, high possibility of lung aspiration, upper respiratory tract infection (URI) symptom, or URI diagnosis within 2 weeks, emergent surgery, or unstable hemodynamic status were excluded from the study.

### Anesthesia protocol

After the pediatric patients arrived in the operating room, standard monitoring such as electrocardiography, non-invasive blood pressure, and pulse oximetry were applied. The pediatric patients were set in the SP using horizontal alignment between the external auditory meatus and the sternal notch, with or without head elevation.

Anesthesia was induced with 5 mg/kg thiopental sodium and 0.6 mg/kg rocuronium through pre-existing intravenous line. Tracheal intubation was performed after three minutes and anesthesia was maintained with inhaled sevoflurane. Prior to using the laryngoscope in the patients, the location to attach the AV scope (CARETEK Co., Ltd, Korea) to the laryngoscope blade was determined using a pediatric mannequin as the area that matched the line of vision of the experimenter and the view of the AV scope. The laryngoscope handle (2.5 v Penlight handle, Welch Allyn®, Inc., USA) was prepared with the AV scope attached, and laryngoscopy was performed with a Macintosh (MAC) blade (#0; length 80 mm or #1; length 87 mm, Welch Allyn®, Inc., USA) inserted into the mouth at the right commissure while pushing the entire tongue to the left of the blade. The laryngeal view was evaluated by lifting the longitudinal axis of the laryngoscope handle with the MAC blade tip pressed against the tongue base (hypoepiglottic ligament)^11^. The percentage of glottis opening (POGO) score was then evaluated through the AV scope screen. Pediatric patients with a POGO score of 100% were excluded from the study, and the patients with a POGO score less than 100% underwent HA-ECTS.

**Figure 1:**
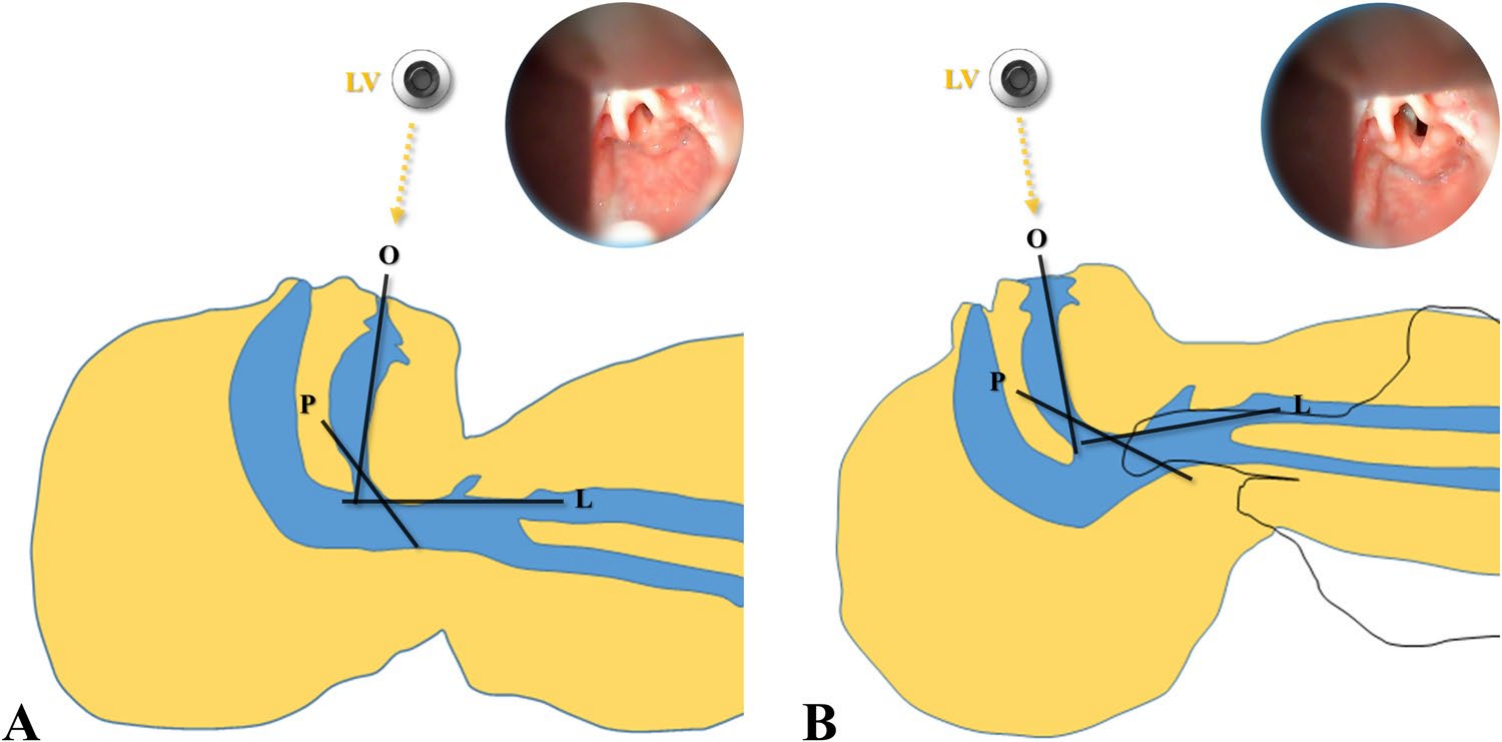
Three axes and line of vision (**A**) before and (**B**) after HA-ECTS. Abbreviations: O, oral axis; P, pharyngeal axis; L, laryngeal axis; LV, line of vision.

The HA-ECTS method was comprised of an assistant performing traction in the upper and caudad directions with both hands under the shoulder (lower cervical area) while supporting the neck with the fingers (Fig. 1). The shoulder was mildly elevated and head extension. After confirming a stable position, POGO score evaluation and tracheal intubation were performed. If tracheal intubation failed with the laryngoscopy, tracheal intubation was performed using a stylet or Glidescope (Verathon Inc., Bothell, WA).

### Outcomes

The POGO, mouth opening (MO), and laryngoscopy handling score (LHS) were measured before and after HA-ECTS. After HA-ECTS, intubation difficulty score (IDS)^12^ and intubation condition score (ICS)^13^ were both measured. The POGO score was assessed twice, the initial POGO score was assessed after 3 minutes of rocuronium administration, and the second POGO score was assessed after applying HA-ECTS. The POGO score was expressed as a percentage of glottis exposure in the laryngoscopy view, which is expressed as 100% for a fully exposed glottis and 0% for an unexposed glottis. Pediatric patients with a POGO score of 100 at the SP were excluded from evaluation by an investigator (JHA) who performed tracheal intubation. Patients with a POGO score less than 100 were evaluated with a captured AV scope screen. After all studies were completed, all images were encoded and randomly assigned, and the POGO scores of the images were evaluated to compare with the figure of standard POGO score by single anesthesiologist who did not participate in the study. MO were measured from upper margin of lower lip to lower margin of upper lip using ruler. LHS evaluated the four categories of mouth opening, teeth contact, sternum contact, and advancement of the laryngoscope (Table 1). The amount of difficulty with laryngoscopic handling was divided into easy (LHS < 2), moderate (2 ≤ LHS < 4), and difficult (LHS ≥ 4 or if there were two points in one item) by summing the scores of the LHS categories. Intubation Difficulty scale(IDS)^12^ includes seven items: number of attempts (every additional attempt adds 1point), number of operator (each additional operator adds 1point), number of alternative technique (each alternative technique adds 1 point), cormack grade (I-0, II-1, III-2 and IV-3 point), lifting force (1 point if subjectively lifting force necessary), external laryngeal pressure (1 point if external laryngeal pressure necessary) and vocal cord mobility (abduction-0 and adduction-1 point). IDS sums the score according to the number of procedures added at the time of intubation and divides it into easy (IDS = 0), slight difficulty (IDS = 1–5), and moderate to severe difficulty (IDS > 5). The intubating condition score(ICS)^13^consists of a total score of 20 in relation to jaw relaxation, laryngoscopy difficulty, limb movement, vocal cord movement and coughing, which are graded as excellent (if score is 5), good (if score is 6–10), poor (if score is 11–15) and bad (if score is 16–20). The primary outcome was change in POGO score after HA-ECTS. The secondary outcomes were the changes in MO and LHS after HA-ECTS.

**Table 1:** Laryngoscopic Handling Score (LHS).

	0	1	2
Mouth opening	No additional mouth opening is required.	Additional mouth opening is required. (with one hand)	Additional mouth opening is required. (with two hand or using tongue depressor)
Teeth contact	The blade does not touch the tooth (or gingiva).	The blade touches the upper or lower teeth (or gingiva).	The blade touches the upper and lower teeth (gingiva).
Sternum contact	The laryngoscope handle can enter without touching the sternum.	The laryngoscope handle can be inserted in a diagonal line.	The laryngoscope handle touches the sternum, preventing entry.
Advancement of laryngoscope	No resistance	There is slight resistance	There is resistance preventing entry.

The ease of laryngoscopic handling was divided into easy (LHS < 2), moderate (2 ≤ LHS < 4), and difficult (LHS ≥ 4 or if there are two points in one item).

### Statistics

Sample size calculations were based on our unpublished pilot study. In a total of 6 pilot studies, the POGO score at the SP was 47 (15), while the POGO score after HA-ECTS was 58 (30). Sample size was calculated using the Wilcoxon signed-rank test. Thirty pediatric patients were required for alpha error 0.05 and power 0.9, and the total sample size was assumed to be 37 patients, assuming a 20% dropout rate. Data are presented as the mean (SD) or median (range) with a 95% confidence interval (CI) as appropriate. Continuous variables were analyzed using paired t-test or Wilcoxon’s signed-rank test, and a normality test was performed using the Shapiro-Wilk test. Categorical variables were analyzed using Pearson’s chi-square test or Fisher’s exact test where appropriate. Subgroup analysis was performed according to age group (0–12 and 12–36 months) and IDS (easy [IDS = 0] and difficult [IDS > 0]). Statistical analyses were performed using SPSS version 22 (SPSS Inc., Chicago, IL, USA). A P-value less than 0.05 was considered to be statistically significant.

## Results

**Figure 2:**
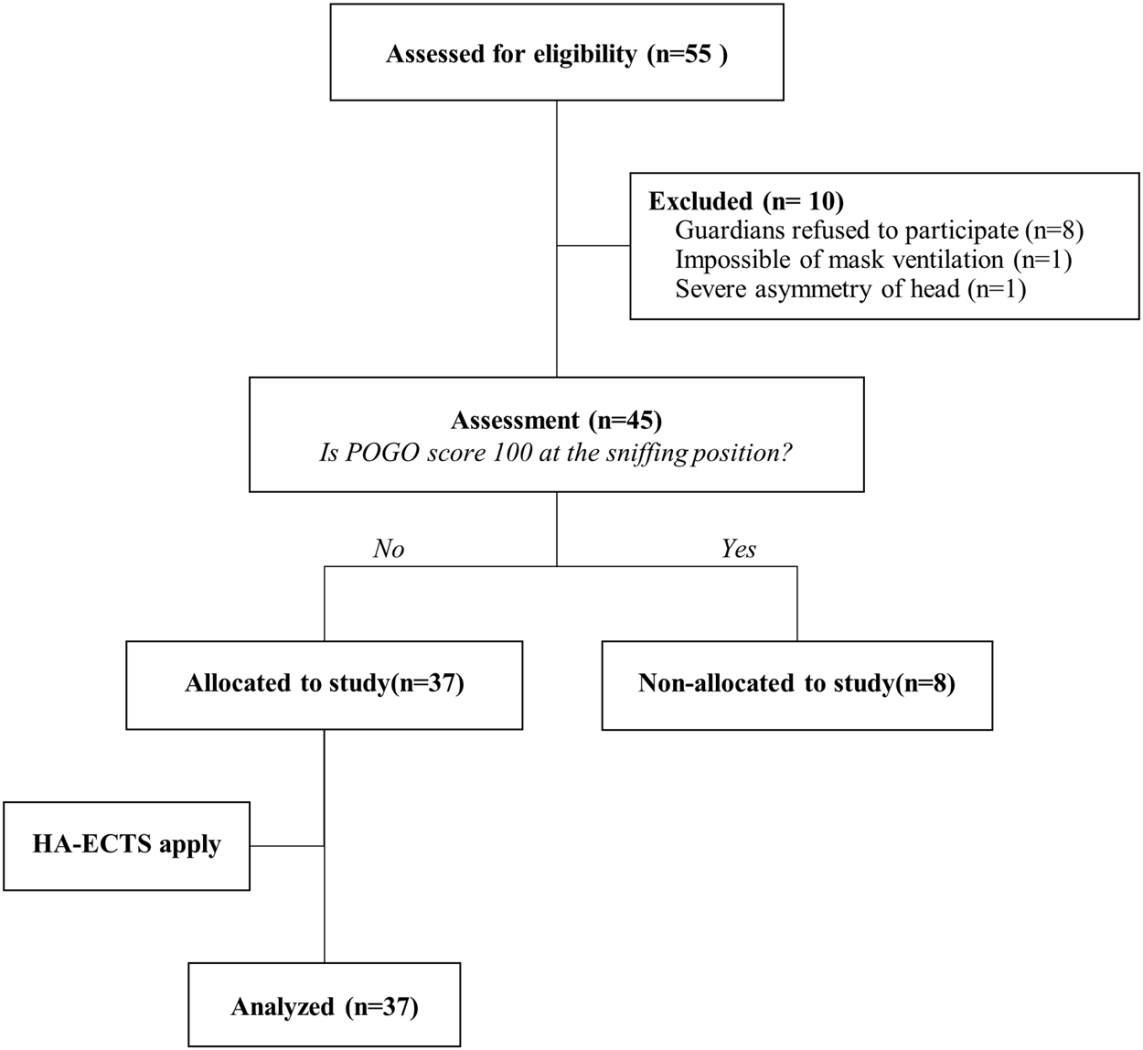
The CONSORT flow diagram.

A total of 55 pediatrics was enrolled for this study (Fig. 2). Of these patients, 10 were excluded for the following reasons: Guardians refused to participate in study (n = 8), impossible mask ventilation (n = 1) and protrusion of the occiput due to hydrocephalus (n = 1). In addition, eight pediatric patients were excluded due to POGO score 100% before HA-ECTS. Therefore, a total of 37 pediatric patients was analyzed.

**Figure 3:**
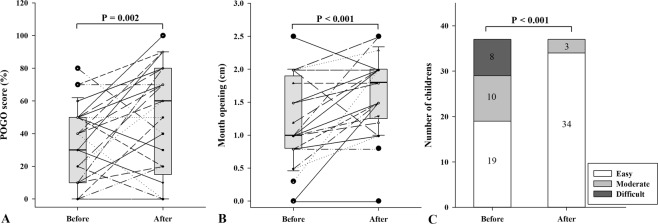
(**A**) POGO score, (**B**) mouth opening, and (**C**) ease of laryngoscopic handling before and after application of HA-ECTS.

Patient characteristics and intubation data are shown in Table 2. The median [range] POGO score was 30 [10–50]% and 60 [15–80]% before and after HA-ECTS, respectively (median difference, 20; 95% CI 10 to 25%; P = 0.002) (Fig. 3A). The median (range) MO was 1.0 [0.8–1.9] cm and 1.8 [1.3–2.0] cm before and after HA-ECTS, respectively (median difference, 0.45 cm; 95% CI 0.25 to 0.60; P < 0.001) (Fig. 3B). Finally, the median (range) LHS was 1.0 [0.0–3.0] and 0.0 [0.0–1.0] before and after HA-ECTS, respectively (median difference, −1.5; 95% CI −1.0 to −2.0%; P < 0.001). The ease of laryngoscopic handling was improved after HA-ECTS (P < 0.001). (Fig. 3C).

**Table 2:** Patient characteristics and intubation data.

	N = 37
Gender (Female/Male)	16/21
Age, months	13.0 [3.5–29.0]
Weight, kg	10.1 [13.0–78.0]
Height, cm	78.0 [63.5–90.3]
ASA PS (I/II/III)	23/8/6
Intubation 1^st^ attempt (Success/Fail)	32/5
Intubation Condition Score^*^, **(n)**	
Excellent	31
Good	6
Poor	0
Bad	0
**Intubation Difficulty Score**^**†**^, **(n)**	
Easy	24
Slight difficulty	12
Moderate to severe difficulty	1

All data are presented as median [range] or number.

^*^The intubating condition score consists of a total score of 20 in relation to jaw relaxation, laryngoscopy difficulty, limb movement, vocal cord movement and coughing, which are graded as excellent (if score is 5), good (if score is 6–10), poor (if score is 11–15) and bad (if score is 16–20).

^†^Intubation Difficulty scale includes seven items: number of attempts (every additional attempt adds 1point), number of operator (each additional operator adds 1point), number of alternative technique (each alternative technique adds 1 point), cormack grade (I-0, II-1, III-2 and IV-3 point), lifting force (1 point if subjectively lifting force necessary), external laryngeal pressure (1 point if external laryngeal pressure necessary) and vocal cord mobility (abduction-0 and adduction-1 point), which are graded as easy (IDS = 0), slight difficulty (IDS = 1–5) and moderate to severe difficulty.

Abbreviations: ASA PS, American Society of Anesthesiologists physical status.

Subgroup analysis before and after HA-ECTS according to age (0–12 and 12–36 months) and IDS (easy [IDS = 0] and difficult [IDS > 0]) is shown in Table 3. After HA-ECTS by age, there was no difference in POGO score in 0–12-month-old children (P = 0.249), but there was significant difference in MO and LHS (P = 0.005 and P = 0.001, respectively). In addition, there was a significant difference in POGO, MO, and LHS in older children aged 12–36 months (P = 0.007, P = 0.003, and P = 0.001, respectively). After HA-ECTS by IDS, there was no difference in POGO score in difficult airways (P = 0.551), but there was significant difference in MO and LDS (P = 0.006 and P = 0.016, respectively). There was also significant difference in POGO score, MO, and LDS in easy airways (P < 0.001, P = 0.002, and P = 0.016, respectively).

**Table 3:** Comparing the POGO score, mouth opening, and ease of laryngoscopic handling before and after HA-ECTS according to age and IDS.

Age (months)	0–12 (n = 18)			12–36 (n = 19)		
	Before	After	P-value	Before	After	P-value
POGO score (%)	35 [0.0–52.5]	45 [0.0–80.0]	0.249	20 [10.0–50.0]	60 [40.0–80.0]	0.007
Mouth opening (cm)	1.0 [0.8–1.6]	1.9 [1.4–2.0]	0.005	1.0 [0.8–2.0]	1.8 [1.2–2.0]	0.003
Ease of laryngoscopic handling (Easy/moderate/difficult)	9/5/4	18/0/0	0001	10/5/4	16/3/0	0.081
**IDS**	**Easy (n = 24)**			**Difficult (n = 13)**		
POGO score (%)	50 [12.5–60.0]	75.0 [52.5–80.0]	<0.001	0.0 [0.0–25.0]^*^	0.0 [0.0–20.0]^†^	0.551
Mouth opening (cm)	1.0 [0.8–2.0]	1.7 [1.1–2.0]	0.002	1.0 [0.8–1.4]	2.0 [1.5–2.0]	0.006
Ease of laryngoscopic handling (Easy/moderate/difficult)	14/6/4	22/2/0	0.016	5/4/4	12/1/0	0.016

Subgroup analysis was performed before and after HA-ECTS according to age (0–12 and 12–36 months) and IDS (easy [IDS = 0] and difficult [IDS > 0]).

All data are presented as median [range] and number.

^*^P = 0.001 versus easy airway.

^†^P < 0.001 versus easy airway.

Abbreviations: POGO, percentage of glottis opening; HA-ECTS, hand-assisted elevation and caudad traction of the shoulder; IDS, intubation difficulty score.

Five pediatric patients (14%) failed intubation on the first attempt, after which intubation with a stylet or glidescope was successful. There were no complications related to the study protocol.

## Discussion

In our study, we found that application of HA-ECTS improved of POGO score, MO, and ease of laryngoscopic handling in pediatric patients younger than 3 years. In patients aged 0–12 months or who had a difficult airway, there were no differences in POGO scores, but MO and ease of laryngoscopic handling improved. In patients aged 12–36 months or with an easy airway, there was improved POGO score, MO, and ease of laryngoscopic handling after HA-ECTS.

In pediatric intubations, we should be aware of the anatomical differences compared to adults. In pediatric patients, the oral cavity is small at birth, but grows in the first year in conjunction with growth of the mandible and teeth. Limited oral cavity and a relatively large tongue are difficult to maneuver around for a laryngoscope due to restriction in the positioning of the tongue during tracheal intubation^14^. In addition, the epiglottis is a long and narrow omega-shaped piece of tissue that must be sufficiently lifted during laryngoscopy to create the appropriate laryngeal view during tracheal intubation^15^. Therefore, in pediatric patients, various methods have been applied to improve laryngoscopic view and intubation conditions^3,11,16,17^. Alignment of the larynx, pharynx, and oral axes (three-axis alignment theory) provide a better laryngoscopic view and intubation condition^3–5^. In previous studies, POGO and laryngoscopic handling scores were improved using pillow adjustment to align the external auditory meatus and the sternal notch in patients aged 3 to 6 years^3^. In pediatric patients, shoulder elevation by shoulder roll is known to be necessary to align the oral, pharyngeal, and laryngeal axes because pediatric patients experience neck flexion due to a prominent occiput in the supine position^1^. In our study, the greatest difference from conventional shoulder elevation was addition of caudad traction. Lee *et al*. reported that the laryngeal axes and line of vision improved by moving the laryngeal structure in the caudal direction with gravity in the back-up position in adults^10^. In pediatric patients, the airway structure is more flexible compared to adults and can easily change with less force. Unlike changes seen from simple repositioning, the forces acting on the axes in an upward and caudal direction were similar to those seen in neck traction. The caudad traction of the shoulder will improve laryngoscopic handling by widening the neck, increasing mouth opening, and restricting neck and head movement. Therefore, the HA-ECTS will improve the laryngoscopic view and laryngoscopic handling by improving alignment of the laryngeal, pharyngeal, and oral axes compared to the SP. Our results support this by demonstrating that POGO score, MO, and ease of laryngoscopic handling were improved after HA-ECTS.

Our study shows that the POGO score was not different after HA-ECTS in 0–12-month-old pediatric patients. Only 9 out of 18 (50%) pediatric patients exhibited an increase in POGO score. For infants, head elevation moves the larynx anteriorly^18^ and glottis opening at the cervical high level (C2/3)^15^. Therefore, HA-ECTS application that manipulates the low cervical to shoulder level may have an effect on the POGO score that does not change in infants or interferes with the line of vision axes of the glottis opening. Improved POGO score was exhibited in pediatric patients aged 12 months or older with glottis opening moving to a low cervical level by improving the line of vision axes.

For pediatric patients with difficult airways in IDS evaluations, improvement in POGO score was not observed. Glottic visualization was more effective with increasing lifting force or external laryngeal pressure than applying HA-ECTS. The more superior location of the larynx in pediatric patients may create difficulty in visualizing laryngeal structures because of the more acute angulation between tongue base and laryngeal opening^14^. In patients with difficult airways, acute angulation between tongue base and laryngeal opening did not improve after HA-ECTS. In addition, iatrogenic injuries, laryngeal edema, and bleeding are more likely to increase as the number of intubation attempts in pediatric patients increases^19,20^. Therefore, intubation using advanced tools such as video-assisted laryngoscope can be safely performed without airway trauma in patients who are difficult to intubate after the first POGO assessment.

One of the advantages of our study is that we segmented and categorized LHS that were not explicitly presented in previous studies. Four subcategories of MO, teeth contact, sternum contact, and advancement of laryngoscopy were used to measure LHS. Therefore, the ease of laryngoscopic handling could be evaluated more objectively. Laryngoscopic insertion, the first step of tracheal intubation, is one of the most important steps of the tracheal intubation process. Pediatric patients are associated with high metabolic demand and low oxygen reserves that shorten the time to significant hypoxemia during apnea^21^. The application of HA-ECTS increased anterior neck space and decreased the contact of the larynx to the sternum as the distance between the mandible and sternum increased, there was no case of sternum contact in all cases. In addition, Mouth opening was spontaneous due to the power of the upward and caudal movements, which eased insertion of the blade and decreased resistance of the blade as the space of the oral cavity increased. Therefore, HA-ECTS may lead to improvement in ease of laryngoscopic handling. In our study, all infants (≤12 months) tended to improve more significantly. LHS of infant (${\rm{\le }}$12 months) were easy after applying HA-ECTS, while moderate difficulty were found in 3 cases after applying HA-ECTS in older ages (12 < months ${\rm{\le }}$36). The airway structure of the infant is more soft and flexible and can cause dynamic obstruction, while the airway control can be easily performed with a minimum manipulation^14,22^. Therefore, HA-ECTS method is clinically useful in younger ages to improve of larygoscopic handling.

The Miller blade is the preferred blade that exposes laryngeal access during tracheal intubation for pediatric patients^9^. However, in patients under 2 years of age, Miller and MAC blades provide similar laryngoscopic views and intubation conditions with either the Miller blade lifting the epiglottis or the Miller and MAC blades lifting the vallecula^11,23^. The MAC blade has the advantage in tracheal intubation because relatively large tongues are swept to one side with the curved blade and does not stimulate the surface beneath the epiglottis^24^. Therefore, we used a MAC blade to perform tracheal intubations in this study.

There are several limitations in our study. First, POGO scores were lower than in previous pediatric airway studies^11,17^. This subjective observation score may have a bias depending on the observer. Therefore, tracheal intubation was performed by a single anesthesiologist using an AV scope to reduce this bias. Second, the height of shoulder elevation was not fixed. Previous methods to support the shoulder roll and head cushion required much preparation and time. However, HA-ECTS has the advantage of instant adjustment to each individual for optimal view. Third, before and after applying HA-ECTS, the laryngeal, pharyngeal, and oral axes were not confirmed by an image study. There is a difference between ideal airway axes and optimized positioning for direct laryngoscopy. Therefore, further studies using imaging are needed to identify the axes.

In conclusion, HA-ECTS improved the POGO scores, MO, and ease of laryngoscopic handling in pediatric patients aged 0 to 36 months. However, patients younger than 12 months with difficult airway only exhibited an improvement in MO and ease of laryngoscopic handling. Therefore, use of HA-ECTS in pediatric patients under 3 years of age may be an assistive technique for tracheal intubations, but may not be effective for patients younger than 12 months of age with difficult airway.

## Supplementary information

Supplement information
