# Supplementary material for: Improvement of laryngoscopic view by hand-assisted elevation and caudad traction of the shoulder during tracheal intubation in pediatric patients
Source: Sci Rep. 2019 Feb 4;9:1174. doi: 10.1038/s41598-018-37770-6 (PMC6362231; doi:10.1038/s41598-018-37770-6)
Supplement: Supplementary file 1 — Supplement information [file 41598_2018_37770_MOESM1_ESM.docx]

**Title: Improvement of laryngoscopic view by hand-assisted elevation and caudad traction of the shoulder during tracheal intubation in pediatric patients.**

**Jin Hee Ahn**^1^**, Doyeon Kim**^1^**, Nam-su Gil**^1^**, Yong Hun Son**^1^**, Bong Gyu Seong**^1^ **and Ji Seon Jeong**^*1^

^1^Department of Anesthesiology and Pain Medicine, Samsung Medical Center, Sungkyunkwan University School of Medicine, Seoul, Korea

JHA and DK contributed equally to this work as first authors:

^*^**Corresponding author:** Ji Seon Jeong, M.D., Ph.D.

Department of Anesthesiology and Pain Medicine, Samsung Medical Center, 81 Irwon-ro, Gangnam, Seoul 06352, Korea

Tel.: +82-2-3410-2463; Fax: +82-2-3410-2461; E-mail: [jiseon78.jeong@samsung.com](mailto:jiseon78.jeong@samsung.com)

**Supplement 1. Standard evaluation figure of POGO score**


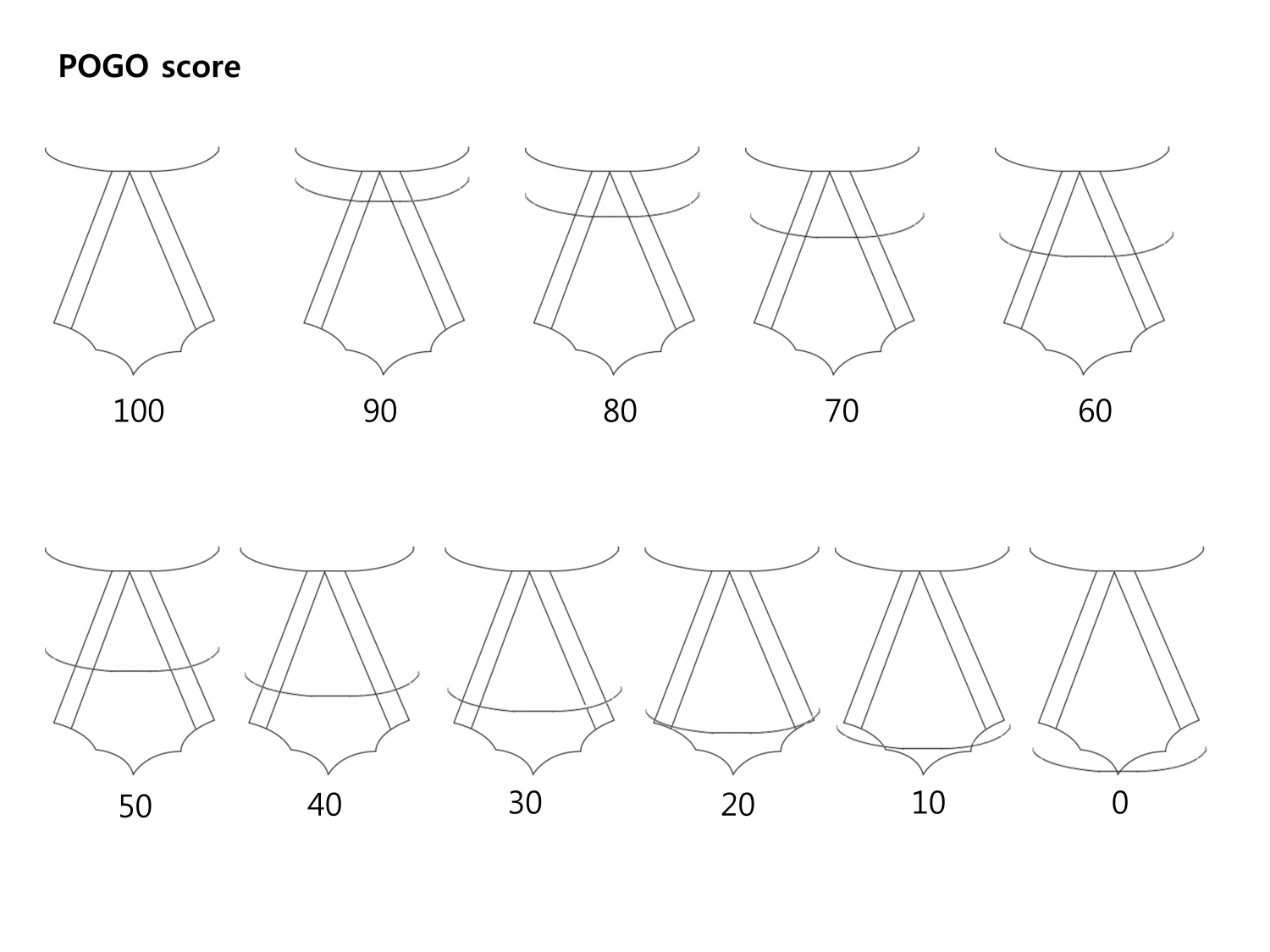


**Supplement 2. Intubation Difficulty Scale**

| **Parameter** | **Score** |
| --- | --- |
| Number of Attempts>1 | N1 |
| Number of Operatiors>1 | N2 |
| Number of alternative techniques | N3 |
| Cormack Grade - 1 | N4 |
| Lifting force required  Normal  Increased | N5=0  N5=1 |
| Laryngeal pressure  Not applied  Applied | N6=0  N6=1 |
| Vocal cord mobility  Abduction  Adduction | N7=0  N7=1 |
| TOTAL IDS = SUM OF SCORES |  |

**●Rules for Calculating IDS score**

- N1 : Every additional attempt adds 1 point

- N2 : Each additional operator adds 1 point

- N3: Each alternative technique adds 1 point. Repositioning of the patient, change of materials(blade, addition of a stylet), change in approach(nasotracheal/orotracheal) or use of another technique (fibroscopy, intubation through a laryngeal mask)

- N4: Apply Cormack grade for 1^st^ oral attempt. For successful blind intubation N4=0

| **IDS score** | **Degree of Difficulty** |
| --- | --- |
| 0 | Easy |
| 0<IDS<5 | Slight Difficulty |
| 5<IDS | Moderate to Severe Difficulty |

- N5: Sellick’s maneuver adds no points

**Supplement 3. Intubation condition scale**

| Points | 1 | 2 | 3 | 4 |
| --- | --- | --- | --- | --- |
| laryngoscopy | Easy | Fair | Difficult | Impossible |
| Vocal cords | Open | Moving | Closing | Closed |
| Coughing | None | Slight | Moderate | Severe |
| Jaw relaxation | Complete | Slight | Stiff | Rigid |
| Limb movements | None | Slight | Moderate | Severe(jerky) |
